# Relative Tissue Factor Deficiency Attenuates Ventilator-Induced Coagulopathy but Does Not Protect against Ventilator-Induced Lung Injury in Mice

**DOI:** 10.1155/2012/130410

**Published:** 2011-12-12

**Authors:** Esther K. Wolthuis, Alexander P. J. Vlaar, Goda Choi, Joris J. T. H. Roelofs, Marcel Levi, Nicole P. Juffermans, Marcus J. Schultz

**Affiliations:** ^1^Department of Anesthesiology, Academic Medical Center, University of Amsterdam, Meibergdreef 9, 1105 AZ Amsterdam, The Netherlands; ^2^Laboratory of Experimental Intensive Care and Anesthesiology (LEICA), Academic Medical Center, University of Amsterdam, Amsterdam, The Netherlands; ^3^Department of Internal Medicine, Academic Medical Center, University of Amsterdam, 1105 AZ Amsterdam, The Netherlands; ^4^Department of Pathology, Academic Medical Center, University of Amsterdam, 1105 AZ Amsterdam, The Netherlands; ^5^Department of Intensive Care Medicine, Academic Medical Center, University of Amsterdam, 1105 AZ Amsterdam, The Netherlands; ^6^HERMES Critical Care Group, Amsterdam, The Netherlands

## Abstract

Preventing tissue-factor-(TF-) mediated systemic coagulopathy improves outcome in models of sepsis. Preventing TF-mediated pulmonary coagulopathy could attenuate ventilator-induced lung injury (VILI). We investigated the effect of relative TF deficiency on pulmonary coagulopathy and inflammation in a murine model of VILI. Heterozygous TF knockout (TF^+/−^) mice and their wild-type (TF^+/+^) littermates were sedated (controls) or sedated, tracheotomized, and mechanically ventilated with either low or high tidal volumes for 5 hours. Mechanical ventilation resulted in pulmonary coagulopathy and inflammation, with more injury after mechanical ventilation with higher tidal volumes. Compared with TF^+/+^ mice, TF^+/−^ mice demonstrated significantly lower pulmonary thrombin-antithrombin complex levels in both ventilation groups. There were, however, no differences in lung wet-to-dry ratio, BALF total protein levels, neutrophil influx, and lung histopathology scores between TF^+/−^ and TF^+/+^ mice. Notably, pulmonary levels of cytokines were significantly higher in TF^+/−^ as compared to TF^+/+^ mice. Systemic levels of cytokines were not altered by the relative absence of TF. TF deficiency is associated with decreased pulmonary coagulation independent of the ventilation strategy. However, relative TF deficiency does not reduce VILI and actually results in higher pulmonary levels of inflammatory mediators.

## 1. Introduction

Mechanical ventilation (MV) is an indispensable tool in the treatment of patients with acute respiratory failure and is mandatory during general anesthesia. However, MV can aggravate preexisting lung injury or even induce lung injury in healthy lungs [1–3]. This phenomenon is frequently referred to as ventilator-induced lung injury (VILI) in animals or ventilator-associated lung injury (VALI) in humans [4]. All mechanically ventilated patients may be at risk for VALI.

Pulmonary coagulopathy is a characteristic feature of acute lung injury [5] and pneumonia [5, 6] and is the result of localized tissue-factor-(TF-) mediated thrombin generation, impaired activity of natural inhibitors of coagulation [7, 8], and depressed bronchoalveolar urokinase plasminogen activator-mediated fibrinolysis, caused by an increase of plasminogen activator inhibitors [9]. Pulmonary fibrin turnover is also influenced by MV [10, 11]. Indeed, in a rat model of thrombin and fibrinogen instillation, induced pulmonary coagulopathy MV with high tidal volumes (*V*_*T*_) attenuated fibrinolytic activity via an increase of local plasminogen activator inhibitors [10]. Similar results were obtained in a model of endotoxin-induced lung injury [11]. In humans, MV during general anesthesia with settings that have been shown to be harmful causes activation of bronchoalveolar coagulation, as reflected in a marked increase in thrombin-antithrombin complexes (TATc), soluble TF, and factor VIIa [12]. Lung-protective ventilator settings largely attenuate these changes in procoagulant activity within the airways.

The direct relation between local TF expression and VILI has never been elucidated. Therefore, the main objective of this study was to determine the role of endogenous TF in alveolar coagulopathy and pulmonary inflammation in response to MV. We hypothesize animals with differences in TF expression to respond in a different way to VILI. For this, we ventilated heterozygous TF knockout (TF^+/−^) mice and their wild-type (TF^+/+^) littermates with either low *V*_*T*_ or high *V*_*T*_.

## 2. Materials and Methods

The study was approved by the Animal Care and Use Committee of the Academic Medical Center of the University of Amsterdam, Amsterdam, The Netherlands. Animal procedures were carried out in compliance with Institutional Standards for Human Care and Use of Laboratory Animals.

### 2.1. Mice

Experiments were performed with heterozygous TF knockout mice (TF^+/−^, *N* = 36) and their wild-type littermates (TF^+/+^, *N* = 36), aged 13–17 weeks. Disruption of the mouse tissue factor gene (TF^−/−^) leads to embryonic lethality [13], while heterozygotes appear normal and free of bleeding complications. TF^+/−^ mouse embryonic fibroblasts have only slightly prolonged clotting times *in vitro*. Schoenmakers have shown that clotting times are very similar in TF^+/−^ mice compared with TF^+/+^ mice, after stimulating with endotoxin [13]. This suggest that TF is dominantly expressed and that lack of the gene in one allele does not affect the coagulation phenotype. TF^+/−^ mice, on a C57Bl/6 background [14], were bred and maintained at the animal care facility of the Academic Medical Center. Sedated but nonventilated mice served as controls (*N* = 12 for either TF^+/−^ or TF^+/+^ mice). Sedated and tracheotomized mice were ventilated with 2 different ventilation strategies (see below). In total this resulted in 6 groups of animals (see below).

### 2.2. Instrumentation and Anesthesia

Throughout the experiments rectal temperature was monitored and maintained between 36.0 and 37.5°C using a warming pad. Anesthesia was achieved with intraperitoneal injection of a mix of ketamine (Eurovet Animal Health BV, Bladel, The Netherlands), medetomidine (Pfizer Animal Health BV, Capelle a/d IJssel, The Netherlands), and atropine (Pharmachemie, Haarlem, The Netherlands) (KMA) as described previously [15, 16]. Induction anesthesia consisted of injection of KMA “induction” mix: 7.5 *μ*L per gram of body weight of 1.26 mL 100 mg/mL ketamine, 0.2 mL 1 mg/mL medetomidine, and 1 mL 0.5 mg/mL atropine in 5 mL normal saline. Maintenance anesthesia consisted of injection of 10 *μ*L per gram body weight of KMA “maintenance” mix, consisting of 0.72 mL 100 mg/mL ketamine, 0.08 mL 1 mg/mL medetomidine, and 0.3 mL 0.5 mg/mL atropine, in 20 mL normal saline. Maintenance mix was administered via an intraperitoneal catheter (PE 10 tubing, BD, Breda, The Netherlands) every hour.

### 2.3. Mechanical Ventilation Strategies

An Y-tube connector, 1.0 mm outer diameter and 0.6 mm inner diameter (VBM Medizintechnik GmbH, Sulz am Neckar, Germany), was surgically inserted into the trachea under general anesthesia. Mice were placed in a supine position and connected to a human ventilator (Servo 900 C, Siemens, Sweden). Mice were pressure-controlled ventilated with either an inspiratory pressure of 10 cm H_2_O (resulting in *V*_*T*_ of about 7.5 mL/kg; lower *V*_*T*_, L*V*_*T*_) *or* an inspiratory pressure of 18 cmH_2_O (resulting in *V*_*T*_ of about 15 mL/kg; higher *V*_*T*_, H*V*_*T*_) as described before [15, 16]. Respiratory rate was set at 110 breaths/min and 50 breaths/min with L*V*_*T*_ and H*V*_*T*_, respectively. These respiratory settings resulted in normal PaCO_2_ values after 5 h of mechanical ventilation [15]. PEEP was set at 2 cm H_2_O with both ventilation strategies. The fraction of inspired oxygen was kept at 0.5. The inspiration to expiration ratio was kept at 1 : 1 throughout the experiment. A sigh (sustained inflation with 30 cm H_2_O) for 5 breaths was performed every 30 minutes. Mice received an intraperitoneal bolus of 1 mL normal saline 1 hour before start of anesthesia and initiation of MV, followed by 0.2 mL sodium bicarbonate (200 mmol/L NaHCO_3_) administered via the intraperitoneal catheter every 30 minutes until the end of MV.

### 2.4. Hemodynamic and Ventilatory Monitoring

Systolic blood pressure and heart rate were noninvasively monitored using a murine tail-cuff system (ADInstruments, Spechbach, Germany). *V*_*T*_ was checked hourly with a specially designed mice “Fleisch-tube” connected to the warmed plethysmograph system. The flow signal was integrated from a differential pressure transducer, and data were recorded and digitized online using a 16-channel data-acquisition program (ATCODAS, Dataq Instruments Inc, ‘s-Hertogenbosch, The Netherlands) and stored on a computer for postacquisition off line analysis. A minimum of 5 consecutive breaths was selected for analysis of the digitized *V*_*T*_ signals.

### 2.5. Study Groups

Nonventilated control mice (TF^+/−^ and TF^+/+^ mice; *N* = 12) received half the dose of anesthesia, were spontaneously breathing, and sacrificed after 5 hours.

L*V*_*T*_ mice and H*V*_*T*_ mice (TF^+/−^ and TF^+/+^ mice) were mechanically ventilated for 5 hours and then sacrificed. Half of these mice (*N* = 6) were sacrificed, and blood was drawn from the inferior vena cava into a sterile syringe, transferred to EDTA-coated tubes, and immediately placed on ice. Subsequently, bronchoalveolar lavage fluid (BALF) was obtained from the right lung; the left lung was used to measure wet/dry ratios. In the other half of these mice (*N* = 6), blood was sampled from the carotid artery and used for blood gas analysis. The lungs of these mice were used for homogenate (right lung) and histopathology (left lung). 

### 2.6. Blood Gas Analysis

For blood gas analysis, blood was immediately analyzed in a Rapidlab 865 blood gas analyzer (Bayer, Mijdrecht, The Netherlands). The other blood samples were centrifuged at 3000 rpm at 4°C for 10 min and the supernatants were aliquoted and frozen at −20°C until assayed.

### 2.7. Lung Wet-to-Dry Ratios

For lung wet-to-dry ratios, the left lung was weighed and subsequently dried for three days in an oven at 65°C. The ratio of wet weight to dry weight represents tissue edema.

### 2.8. Lung Homogenates

During sacrificing the right lung was removed and snap frozen in liquid nitrogen. These frozen specimens were suspended in 4 volumes of sterile isotonic saline, subsequently lysed in 1 volume of lysis buffer (150 mM NaCl, 15 mM Tris (tris(hydroxymethyl)aminomethane), 1 mM MgCl·H_2_O, 1 mM CaCl_2_, 1% Triton X-100, 100 *μ*g/mL pepstatin A, leupeptin and aprotinin, pH 7.4), and incubated at 4°C for 30 min. Homogenates were spun at 3400 rpm at 4°C for 15 minutes after which the supernatants were stored at −20°C until assayed.

### 2.9. Bronchoalveolar Lavage

BALF was obtained by instilling three times 0.5 mL aliquots of saline into the right lung by a 22-gauge Abbocath-T catheter (Abbott, Sligo, Ireland). Approximately, 1.0 mL of lavage fluid was retrieved per mouse, and cell counts were determined using a hemacytometer (Beckman Coulter, Fullerton, CA, USA). Subsequently, differential counts were done on cytospin preparations stained with a modified Giemsa stain, Diff-Quick (Dade Behring AG, Düdingen, Switzerland). Supernatant was stored at −80°C for total protein level, thrombin-antithrombin complexes, and levels of plasminogen activator inhibitor (PAI)-1.

### 2.10. Histopathology

For histopathology lungs were fixed in 4% formalin and embedded in paraffin. 4 *μ*m sections were stained with hematoxylin-eosin (H&E), and analyzed by a pathologist who was blinded for group identity. To score lung injury, we used a modified VILI histology scoring system as previously described [17]. In short, four pathologic parameters were scored on a scale of 0–4: (a) alveolar congestion, (b) hemorrhage, (c) leukocyte infiltration, and (d) thickness of alveolar wall/hyaline membranes. A score of 0 represents normal lungs; (1) mild, <25% lung involvement; (2) moderate, 25–50% lung involvement; (3) severe, 50–75% lung involvement; (4) very severe, >75% lung involvement. The total histology score was expressed as the sum of the score for all parameters.

### 2.11. Assays

Total protein levels in BALF were determined using a Bradford Protein Assay Kit (OZ Biosciences, Marseille, France) according to manufacturers' instructions with bovine serum albumin as standard. Cytokine and chemokine levels in lung homogenates were measured by enzyme-linked immunosorbent assay (ELISA) according to the manufacturer's instructions. Tumor necrosis factor alpha (TNF), interleukin-(IL-) 6, macrophage inflammatory protein (MIP)-2 and keratinocyte-derived chemokine (KC) assays were all obtained from R&D Systems (Abingdon, UK). Thrombin-antithrombin complex levels in BALF were measured with a mouse specific ELISA as previously described [18]. Levels of PAI-1 were measured by means of a commercially available ELISA (Kordia, Leiden, The Netherlands).

### 2.12. Statistical Analysis

All data in the results are expressed as means ± SEM or median ± interquartile range, where appropriate. To detect differences between groups, the Dunnett method, in conjunction with two-way analysis of variance, was used. A *P*-value of <0.05 was considered significantly. All statistical analyses were carried out using SPSS 12.0.2 (SPSS, Chicago, IL, USA).

## 3. Results

### 3.1. Hemodynamic and Ventilatory Monitoring

**Figure 1:**
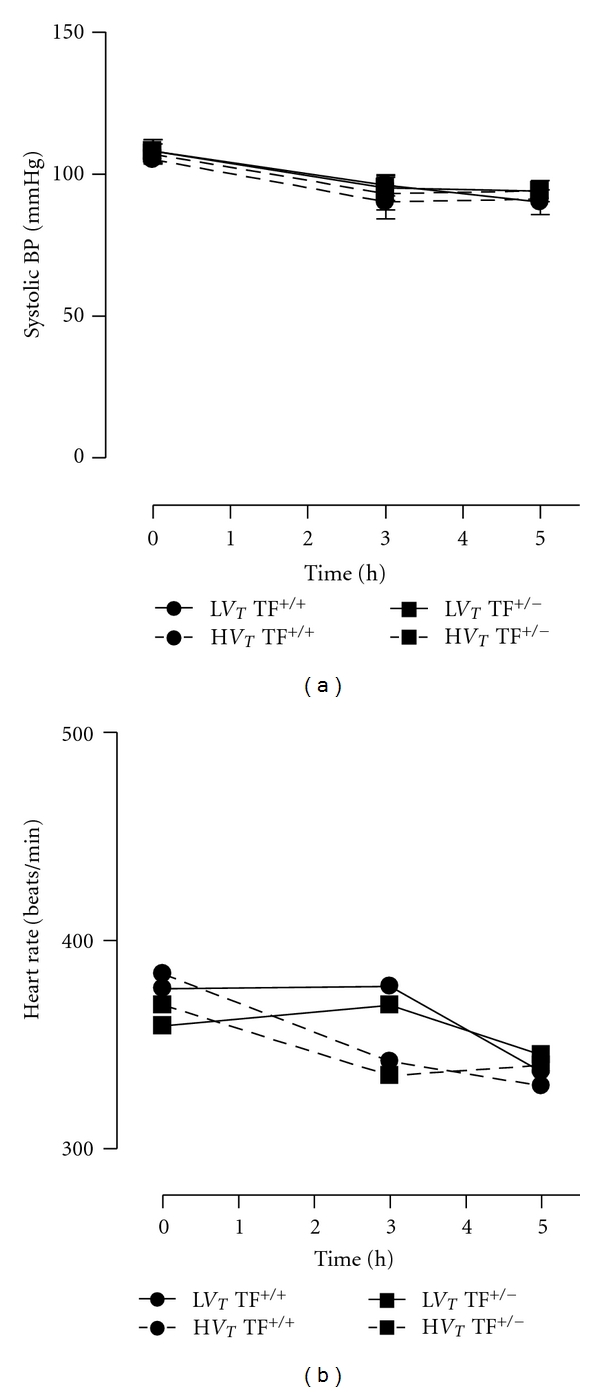
Hemodynamic parameters during 5 hours of mechanical ventilation. Systolic blood pressure (a) and heart rate ((b); beats/min) were measured at three time points (*t* = 0, 2.5, and 5 hours after start of mechanical ventilation) in mice ventilated with low tidal volumes (L*V*_*T*_) and high tidal volumes (H*V*_*T*_). Each mentioned group consisted of heterozygote TF knockout (TF^+/−^) mice and their wild-type littermates (TF^+/+^). Data represent means of 12 mice.

All instrumented animals survived 5 hours of MV after which they were sacrificed. Control animals survived anesthesia and were also sacrificed after 5 hours. Hemodynamic monitoring demonstrated stable conditions throughout the experiment with no differences between TF^+/−^ and TF^+/+^ mice and ventilation groups (Figure 1). Blood gas analysis from L*V*_*T*_-mice and H*V*_*T*_-mice are demonstrated in Table 1. No differences were observed between groups, except from arterial oxygen tension (PaO_2_), which was significantly higher in H*V*_*T*_-mice as compared to L*V*_*T*_-mice.

### 3.2. Ventilator-Induced Pulmonary Coagulopathy

**Figure 2:**
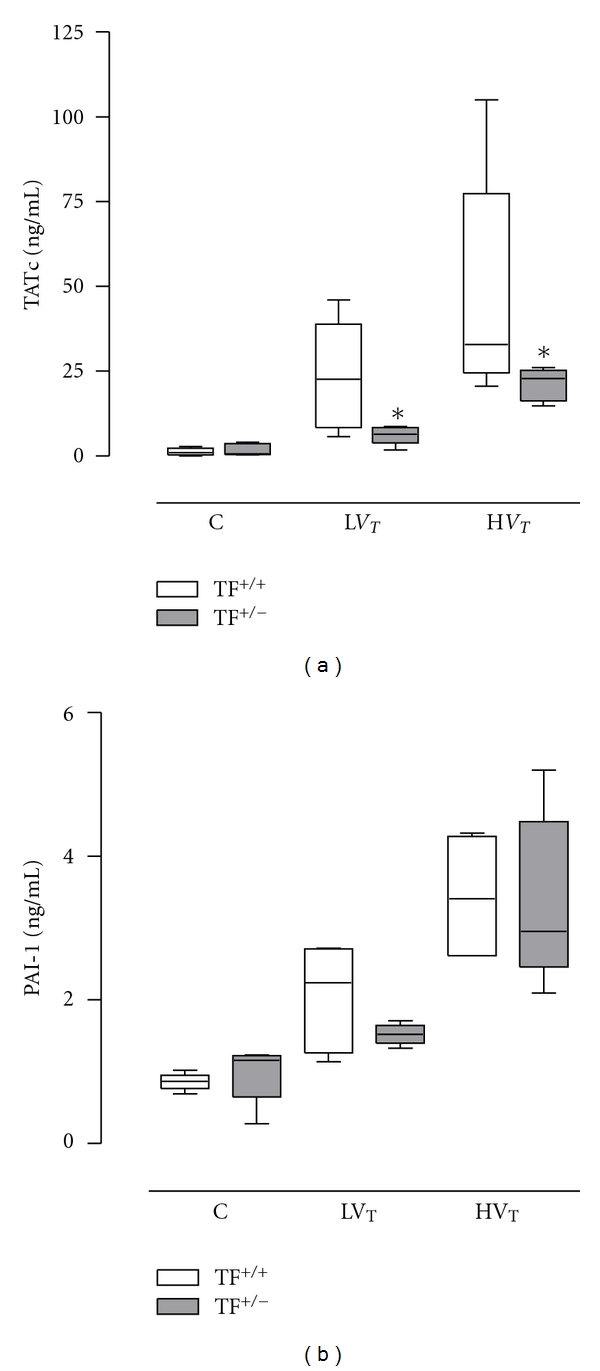
Bronchoalveolar lavage fluid thrombin-antithrombin complex (TATc) and plasminogen activator inhibitor (PAI)-1 levels in control mice (C) and mice ventilated for 5 hours with lower tidal volumes (L*V*_*T*_) or higher *V*_*T*_ (H*V*_*T*_). Each group consisted of heterozygote TF knockout mice (TF^+/−^) and their wild-type littermates (TF^+/+^). Data represent medians and interquartile range of six mice. *P < 0.05 versus TF^+/+^ mice.

BALF TATc levels were higher in both ventilation groups as compared to control mice (*P* = 0.006 for L*V*_*T*_-mice and *P* < 0.001 for H*V*_*T*_-mice), with higher levels in H*V*_*T*_-mice (*P* < 0.001 versus L*V*_*T*_-mice; Figure 2). Compared with TF^+/+^ mice, TF^+/−^ mice demonstrated lower BALF TATc levels in both ventilation groups (*P* = 0.04 and *P* = 0.0026 in L*V*_*T*_-mice and H*V*_*T*_-mice, resp.).

 BALF PAI-1 levels were higher in both ventilation groups as compared to control mice (*P* = 0.001 for L*V*_*T*_-mice and *P* < 0.001 for H*V*_*T*_-mice), with higher levels in H*V*_*T*_-mice (*P* < 0.007 versus L*V*_*T*_-mice; Figure 2). No differences were observed between TF^+/+^ and TF^+/−^ mice regarding BALF PAI-1 levels.

### 3.3. Lung Wet-to-Dry Ratio, Total Protein and Neutrophils in BALF

**Figure 3:**
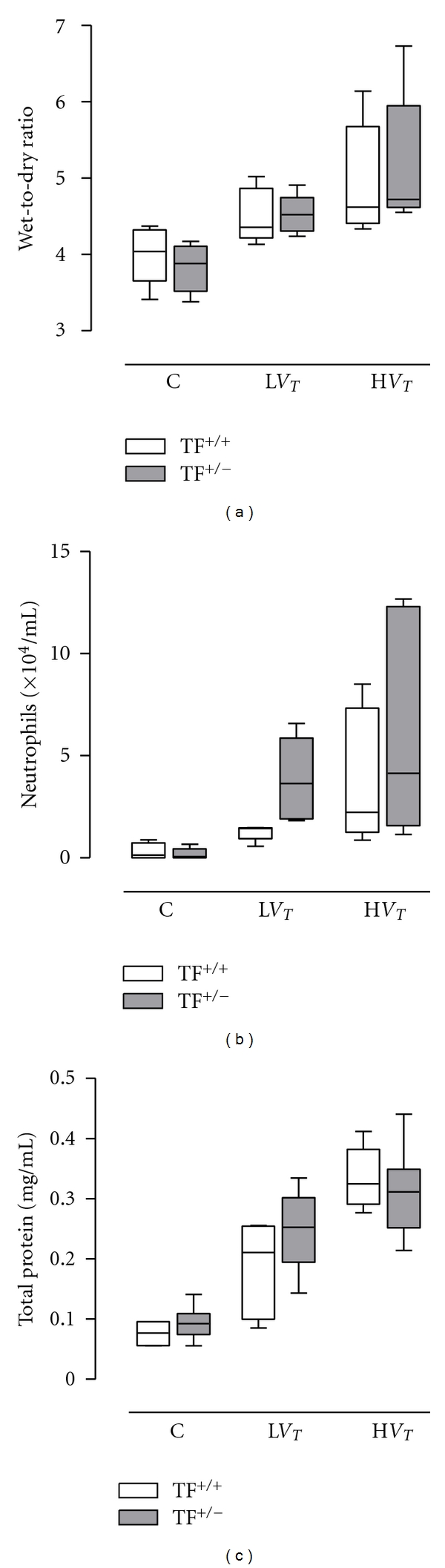
Wet-to-dry ratios, number of neutrophil cells, and total protein level in bronchoalveolar lavage fluid after 5 hours in control mice (C) and mice ventilated for 5 hours with lower tidal volumes (L*V*_*T*_) or higher VT (H*V*_*T*_). Each group consisted of heterozygote TF knockout mice (TF^+/−^) and their wild-type littermates (TF^+/+^). Data represent medians and interquartile range of six mice. Differences between TF^+/−^ and TF^+/+^ are not statistically significant.

Compared with L*V*_*T*_-mice and control mice, lung wet-to-dry ratios were higher in H*V*_*T*_-mice (*P* = 0.039 and *P* < 0.001, resp.; Figure 3). In accordance, BALF total protein levels were higher in H*V*_*T*_-mice as compared to L*V*_*T*_-mice and control mice (*P* < 0.001 and *P* < 0.001, resp.; Figure 3). Also the numbers of neutrophils in the BALF were higher in H*V*_*T*_-mice as compared to control mice (*P* < 0.001), and there was a trend of higher numbers of neutrophils in L*V*_*T*_-mice as compared to control mice (*P* = 0.059; Figure 3). No differences were seen between TF^+/−^ and TF^+/+^ mice with respect to lung wet-to-dry ratio, total protein and neutrophils in BALF.

### 3.4. Lung Histopathology

**Figure 4:**
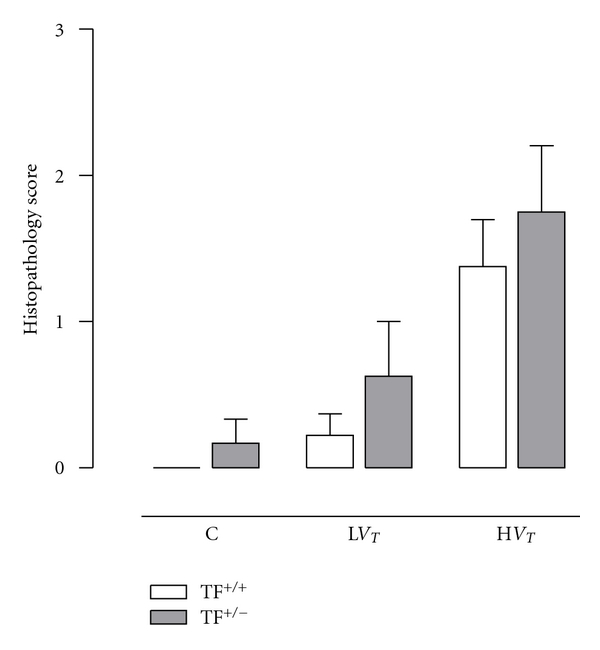
Histopathology scores in control mice (C) and mice ventilated for 5 hours with lower tidal volumes (L*V*_*T*_) or higher VT (H*V*_*T*_). Each group consisted of heterozygote TF knockout mice (TF^+/−^) and their wild-type littermates (TF^+/+^). Data represent means and SEM of six mice. Differences between TF^+/−^ and TF^+/+^ are not statistically significant.

**Figure 5:**
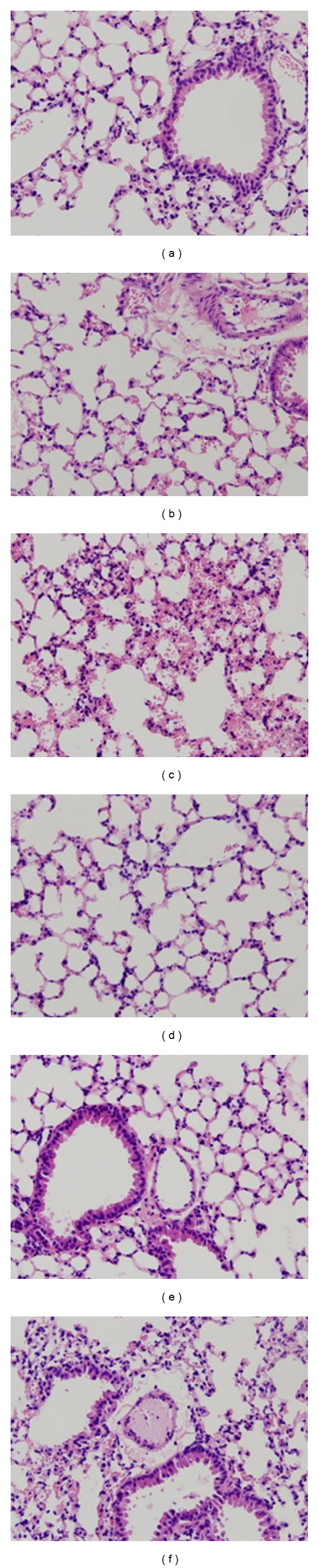
Histological specimens from lungs of control mice (a and d) and mice ventilated with lower tidal volumes (L*V*_*T*_; b and e) or higher *V*_*T*_ (H*V*_*T*_; c and f) for 5 hours. Upper panel, represent wild-type mice (TF^+/+^) and lower panel, represent heterozygote TF knock-out mice (TF^+/−^). Hematoxylin-eosin stain; magnification 200×.

Histopathological changes were minor (Figures 4 and 5). None of the lungs showed signs of inflammation, congestion or hyaline membranes. Only hemorrhage could be seen. The pulmonary histopathology score was higher in H*V*_*T*_-mice as compared to L*V*_*T*_-mice and control mice (*P* = 0.001 and *P* < 0.001, resp.). No differences in histopathology were seen between TF^+/−^ mice and TF^+/+^ mice.

### 3.5. Inflammatory Mediators

**Figure 6:**
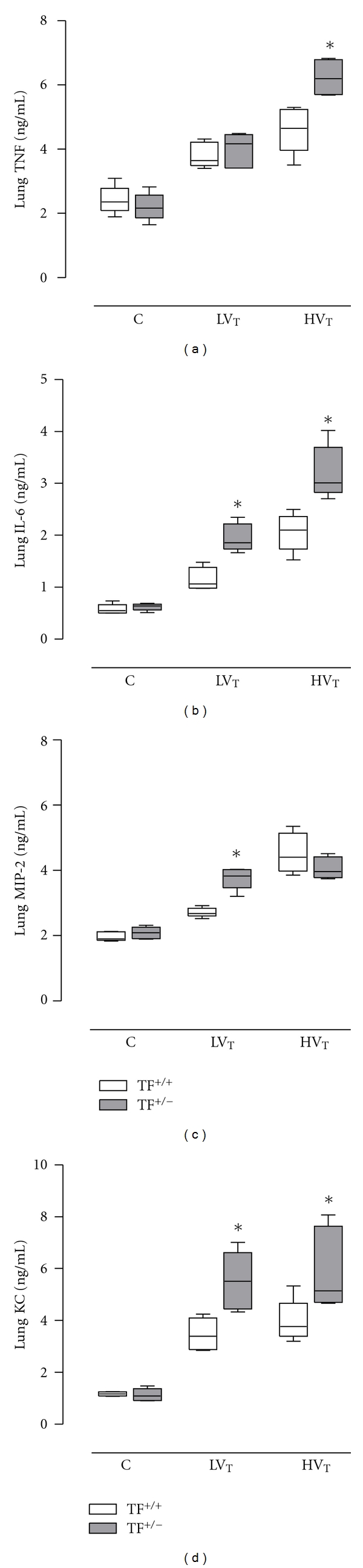
Pulmonary levels tumor necrosis factor alpha (TNF), interleukin (IL)-6, macrophage inflammatory protein (MIP)-2, and keratinocyte-derived chemokine (KC) in lung tissue homogenate in control mice (C) and mice ventilated for 5 hours with lower tidal volumes (L*V*_*T*_) or higher VT (H*V*_*T*_). Each group consisted of heterozygote TF knock-out mice (TF^+/−^) and their wild-type littermates (TF^+/+^). Data represent medians and interquartile range of six mice. *P < 0.05 versus TF^+/+^ mice.

H*V*_*T*_-mice consistently demonstrated higher pulmonary levels of TNF, IL-6, MIP-2 and KC as compared to L*V*_*T*_-mice and control mice (for all mediators *P* < 0.001 and *P* < 0.001, resp.; Figure 6). Compared to TF^+/+^ mice, TF^+/−^ mice had higher levels of IL-6 in both ventilation groups (*P* = 0.002 for L*V*_*T*_ mice and H*V*_*T*_ mice, resp.), and levels of TNF were higher in TF^+/−^ mice as compared to TF^+/+^ mice ventilated with H*V*_*T*_ (*P* = 0.002). Similarly, TF^+/−^ mice demonstrated higher levels of MIP-2 (compared with ventilation with L*V*_*T*_, *P* = 0.002) and KC (*P* = 0.002 and *P* = 0.015 compared with L*V*_*T*_-mice and H*V*_*T*_-mice, resp.).

**Figure 7:**
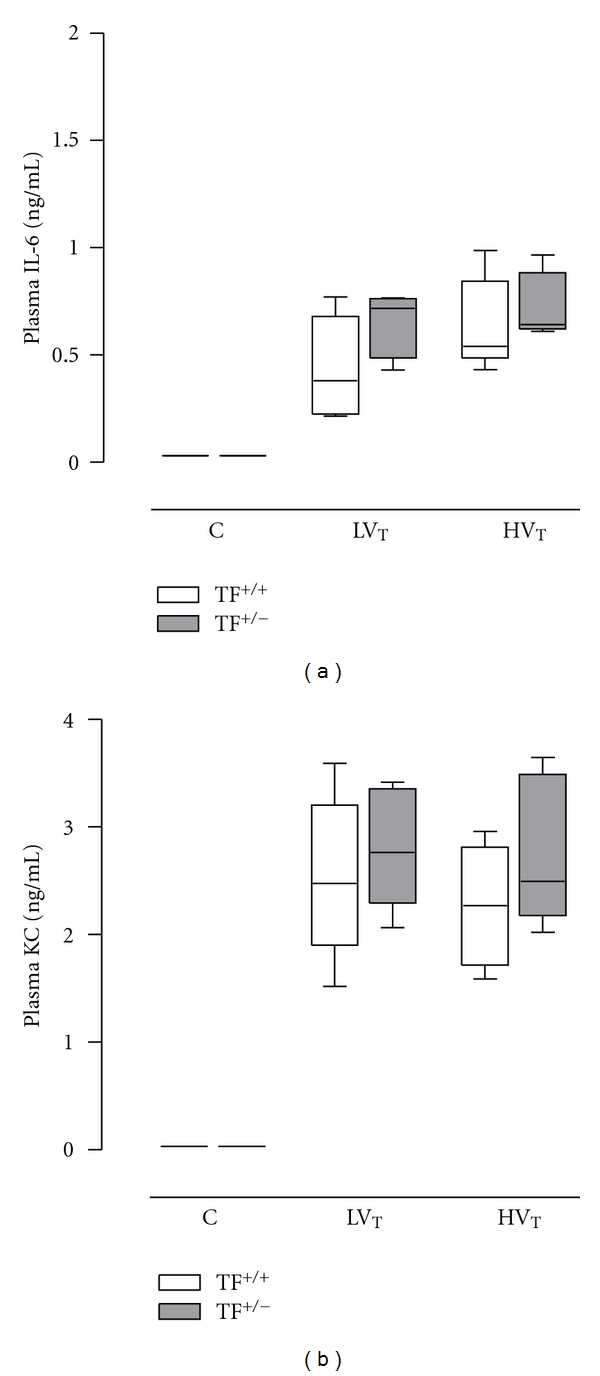
Systemic levels of interleukin (IL)-6 and keratinocyte-derived chemokine (KC) in plasma after 5 hours in control mice (C) and mice ventilated for 5 hours with lower tidal volumes (L*V*_*T*_) or higher VT (H*V*_*T*_). Each group consisted of heterozygote TF knock-out mice (TF^+/−^) and their wild-type littermates (TF^+/+^). Data represent medians and interquartile range of six mice. Differences between TF^+/−^ and TF^+/+^ are not statistically significant.

 Systemic levels of TNF and MIP-2 were below detection limits. Systemic levels of IL-6 and KC were elevated in both ventilation groups as compared to control (*P* < 0.001 and *P* < 0.001 for L*V*_*T*_ mice and H*V*_*T*_ mice, resp.). No differences were seen between TF^+/−^ and TF^+/+^ mice (Figure 7).

## 4. Discussion

There is mounting evidence indicating that TF plays a pivotal role in the procoagulant response in the pulmonary compartment during various forms of pulmonary inflammation and lung injury [19–21]. The present data show TF also to be associated with pulmonary ventilator-induced coagulopathy. Indeed, relative TF deficiency attenuated pulmonary ventilator-induced activation of coagulation, while leaving ventilator-induced inhibition of fibrinolysis unchanged. Relative TF deficiency, however, did not attenuate VILI. In fact, relative TF deficiency induced more inflammation, as reflected by higher pulmonary levels of proinflammatory cytokines and chemokines. Our study is among the first to investigate the potential role of TF in ventilator-induced inflammation.

Because of extensive crosstalk between coagulation and inflammation, causing reciprocal activation and amplification, one may expect relative TF deficiency-related attenuation of ventilator-induced pulmonary coagulopathy to be associated with less pulmonary inflammation. This was indeed found in a murine model of intestinal ischemia-reperfusion-induced lung injury [22]. In human TF knock-in transgenic mice, He et al. demonstrated that administration of an anti-human TF monoclonal antibody (mAb) attenuates severity of lung tissue injury and decreases total cell counts and protein concentrations in BALF. In addition, treatment with anti-human TF mAb resulted in reduced alveolar fibrin deposition and decreased TF and PAI-1 activity in serum. Pulmonary levels of inflammatory cytokines and cell death were also reduced by treatment of anti-human TF mAb. In a rat model of lung injury induced by LPS, blockade of the TF-FVIIa complex protects the lung from injury in part by reducing local expression of proinflammatory cytokines [23]. The differences found in these studies and ours could be explained, at least in part, by the fact that genetic background can influence the different responses of animals to challenges.

Several studies, however, show inhibition of the TF pathway not to dampen inflammation. Indeed, in an *Escherichia coli* peritonitis mice model, treatment with rNAPc2, a potent and selective small protein inhibitor of the TF-pathway, attenuated the local procoagulant response but did not attenuate inflammation [24]. In addition, in a *Streptococcus pneumoniae* pneumonia model in mice, while rNAPc2 treatment attenuated the procoagulant response, it increased pulmonary levels of KC and MIP-2, without influencing clearance of bacteria [25]. Our findings are in agreement with these results, showing higher levels of inflammatory mediators in the pulmonary compartment after MV in TF^+/−^ mice as compared to TF^+/+^ mice.

Our results indicate that differences in TF expression are associated with differences in ventilator-induced coagulopathy. As expected, relative TF deficiency resulted in attenuation of activation of coagulation, while leaving fibrinolysis unchanged. Inactivation of the TF pathway, thus, may lead to less coagulation and less fibrin deposition in the airways during MV. On the other hand, less TF activity leads to more pulmonary inflammation in our VILI model after 5 hours of MV. It can be hypothesized that the cytokine levels in TF^+/+^ mice peaked earlier and that the mice with a relative TF deficiency show a slower pulmonary cytokine response. In a recently published paper of Hegeman and colleagues, they show that the cytokine peak in the lung is after two hours of MV in healthy mice ventilated with a peak inspiratory pressure of 20 cm H_2_O and 0 cm H_2_O PEEP [26]. Adhesion molecules, cytokines, and chemokines are all upregulated in organs distal to the lung, such as liver and kidney due to alveolar stretch imposed by MV in this last mentioned study. Endothelial activation and inflammation in the lung and distal organs are likely to play a role in the various interrelated processes that lead to VILI and other MV-related complications [27, 28], such as multiorgan dysfunction syndrome and possibly ventilator-associated pneumonia.

VILI can exacerbate existing lung injury or make the lung more sensitive to further injury (so-called two-hit model), which is significant with large transfusions, large fluid shifts, cardiopulmonary bypass, and lung ischaemia-reperfusion injury. VILI involves a complicated interaction of overdistension of alveoli, increased transpulmonary pressure, cycling opening, and closing of alveoli and inflammatory mediators. Mechanotransduction is the link between the physical forces imposed on the lung and intracellular signaling pathways leading to the production of cytokines and other biomarkers. Shear stress induces proinflammatory cytokines, mostly by upregulation of nuclear factor (NF)-*κ*B in endothelial, epithelial, and macrophage cells and induces a procoagulant state in the pulmonary compartment. It remains the question if pulmonary coagulopathy promoting fibrin depositions in the airways reflects an adaptive mechanism with host protective function or whether it is a harmful process predisposing the lungs to more complications.

One shortcoming of our study lies in the use of genetically modified animals. The genetic background of transgenic mice can influence different responses. Indeed, when we knock out or knock down a gene from the genome, it may trigger compensatory mechanisms. The responses of transgenic animals to a challenge, thus, reflect the dysfunction (or alteration of the function) of the gene of interest plus compensatory mechanisms. Therefore, the lack of difference between TF^+/−^ and TF^+/+^ mice cannot exclude the possibility of using anti-TF therapies for VILI. Additional studies are needed to test this hypothesis.

Our model has several limitations. First, MV with L*V*_*T*_ could promote development of atelectasis. In our model we used deep inflation every 30 minutes to prevent atelectasis. Periodic deep inflation can open the lung but, if delivered too frequently, may cause damage via repeated overdistension by itself [29]. It may also be speculated that the lungs were not recruited frequently enough to prevent atelectasis in our model. Indeed, H*V*_*T*_ mice demonstrated higher PaO_2_ levels than L*V*_*T*_ mice. Some pulmonary inflammation seen in L*V*_*T*_ mice may have been caused by atelecttrauma. Alternatively, even MV using a *V*_*T*_ of 7.5 mL/kg could still cause overdistension of alveoli. Second, the *V*_*T*_ used in H*V*_*T*_ mice is quite large. Although there is still underuse of lung-protective ventilation with the use of lower *V*_*T*_ [30, 31], *V*_*T*_ has declined gradually over the past 10 years [32, 33]. Another limitation is that lung injury induced by MV in our model was generally small. It remains to be determined (a) whether relative TF deficiency is more protective, in respect to lung injury with more injurious forms of MV, and (b) whether relative TF deficiency protects against VILI in models in which animals were first primed with injured lungs (e.g., after a first hit with lipopolysaccharide or pneumonia).

## 5. Conclusions

In conclusion, TF deficiency is associated with a decreased pulmonary coagulation independent of the ventilation strategy in a murine model of MV. However, relative TF deficiency does not reduce VILI and even results in higher pulmonary levels of inflammatory mediators.

## Figures and Tables

**Table 1:** Arterial blood gas analysis after 5 hours of mechanical ventilation.

	L*V*_*T*_		H*V*_*T*_	
	TF^+/−^	TF^+/+^	TF^+/−^	TF^+/+^
pH	7.44 ± 0.08	7.45 ± 0.06	7.44 ± 0.07	7.44 ± 0.08
PaCO_2_ (mmHg)	35 ± 10	34 ± 8	33 ± 8	33 ± 8
PaO_2_ (mmHg)	165 ± 39	172 ± 48	236 ± 24^†^	234 ± 26*
HCO_3_^−^ (mmol/L)	22.7 ± 2.9	22.6 ± 2.3	22.5 ± 2.1	21.1 ± 1.9
BE	−0.8 ± 2.7	−0.5 ± 0.7	0.8 ± 2.1	−2.1 ± 1.8

Data represent mean ± SD; L*V*_*T*_: mice ventilated for 5 h with *V*_*T*_ of 7.5 mL/kg; H*V*_*T*_: mice ventilated for 5 h with *V*_*T*_ of 15 mL/kg. TF^+/−^: heterozygous TF knockout mice; TF^+/+^: wild-type littermates; *N* = 6 per group. *P < 0.05; ^†^ *P* < 0.01 versus L*V*_*T*_ ventilation.
